# Effect of Agroforestry and Cocoa-Producing Geographical Origin on the Sensory Profile of Beans and Chocolates in the Climate Change Context in Côte d’Ivoire

**DOI:** 10.3390/foods14244321

**Published:** 2025-12-15

**Authors:** Florent G. Kouamé Amien, Maï Koumba Koné, Christian Adobi Kadjo, Alfred Koffi Yao, Isabelle Maraval, Renaud Boulanger, Simplice Tagro Guéhi

**Affiliations:** 1 Unité de Formation et de Recherche Sciences et Technologies des Aliments, Université Nangui ABROGOUA, 02 BP 801, Abidjan 02, Côte d’Ivoire; abaamien1er@gmail.com (F.G.K.A.); alfredyao94@gmail.com (A.K.Y.); 2 Institut National Polytechnique Félix Houphouët-Boigny, Yamoussoukro Bp 1093, Côte d’Ivoire; mahikoumba@yahoo.fr; 3 Unité de Formation et de Recherche Sciences et Technologies, Université Alassane Ouattara, Bouaké BP V 18 01, Côte d’Ivoire; adobik1@gmail.com; 4 CIRAD, UMR Qualisud, TA B 96/16. 75, Rue Jean-François Breton, 34398 Montpellier, Cedex 5, France; isabelle.maraval@cirad.fr (I.M.); renaud.boulanger@cirad.fr (R.B.); 5 Qualisud, Univ Montpellier, CIRAD, Université d’Avignon, Université de la Réunion, Montpellier SupAgro, Montpellier 1101, Avenue Agropolis, 34090 Montpellier, France

**Keywords:** agroforestry, cocoa beans, chocolate, volatile compounds, Côte d’Ivoire

## Abstract

This paper investigated the effects of agroforestry (AF) on the sensory profiles of cocoa beans and the organoleptic quality of end-chocolates. A three-day opening delay for the Ivorian hybrid cultivar commonly known as “Mercedes” (Amelonado × West African Trinitario) from AF and full-sun (FS) plantations as control located at five cocoa-producing areas were fermented in wooden boxes for 6 days and stirred at days 2 and 4. Fermented cocoa was sun-dried until reaching 7–8% moisture and processed into chocolate. Volatile compounds of cocoa powder and chocolate were analyzed using the SPME-GC-MS method, while the organoleptic perception of chocolates was assessed by 12 professional judges according to 10 sensory descriptors. The findings revealed that the concentrations of esters ranged from 9.41 ± 0.61 to 19.35 ± 1.28 µg.g^−1^, aldehydes from 11.56 ± 0.7 to 25.33 ± 1.5 µg.g^−1^, and ketones from 5.76 ± 0.62 to 55.84 ± 4.39 µg.g^−1^ in cocoa beans regardless of the cropping system. However, the concentrations of some volatile compounds classes including alcohols, acids, and pyrazines were similar in AF and FS chocolate samples. AF system clearly influenced the volatile compound profiles of cocoa beans in only the Adzopé, Guibéroua, and Méagui regions without impacting those of the chocolates regardless of the geographical origin after fermentation and roasting. Furthermore, AF chocolate was not less appealing than the FS chocolate samples. So, AF system did not significantly influence the sensory perception of chocolate. AF can therefore be encouraged as a cropping system for cocoa cultivation to reduce deforestation and promote reforestation, ensuring the sustainability of cocoa.

## 1. Introduction

Cocoa beans and chocolate are known as luxury foods that provide an astringent taste and typical aroma [1]. Cocoa, a perennial crop highly cultivated only in the equatorial regions, holds significant economic importance in several countries including Côte d’Ivoire and Ghana [2], accounting for 70% of the cocoa international supply [3], providing incomes for 2 million farmers. Unfortunately, both countries are incriminated in deforestation while benefiting from cocoa production [4]. Cocoa bean is the main raw material for chocolate and other cocoa products [5]. Chocolate is one of the most consumed foods worldwide due to its unique sensory and sensory characteristics, resulting from the unique and fascinating cocoa sensory [6,7]. The quality parameters of the finished chocolate are strongly influenced by cocoa farmers’ farming practices at the start of the chocolate supply chain [8]. The chocolate’s sensory quality largely depends on the genotype, the soil quality, the microclimatic variables, as the primary postharvest processing and the industrial process of beans until obtaining chocolate, at which point the fermentation and the roasting are emphasized [6,9,10,11]. Various studies and surveys showed differences in the farming practices regarding cocoa growing between farmers within the same country [8]. However, several studies emphasize that the primary postharvest processing of cocoa, such as cocoa pod opening delay, fermentation, and drying carried out mainly by farmers, must target the production of specialty cocoa, and the processing conditions must be controlled by integrating the quality characteristics required by the chocolate market [6,9]. The postharvest processing of cocoa is mediated by dynamic biochemical reactions under the actions of successive microorganisms including yeasts, lactic acid bacteria, and acetic acid bacteria spontaneously inoculating cocoa pulp and producing ethanol and lactic and acetic acids [6,12]. Endogenous enzymes catalyze the production of peptides and amino acids from seed storage proteins [13], and the inversion of sucrose and the subsequent formation of reducing sugars occur [14]. The resulting metabolites from enzymatic reactions of proteolysis and hydrolysis of the biochemical seed components are volatile compound precursors [6,15]. Then, during roasting, these products interact through nonenzymatic browning Maillard reactions, leading to the generation of molecules including pyrazines, alcohols, ketones, aldehydes, and esters. In turn, these molecules are responsible for the final sensory notes comprising the chocolate sensory attributes, e.g., flowery, fruity, caramel, nutty, etc. [15]. Thus, the sensory profiles of cocoa beans and of chocolate depend on the biochemical composition of the fresh cocoa pulp [16,17,18]. Cocoa plantations are one of many drivers of deforestation, and assessments must consider competing sectors in a landscape [19]. Several inconveniences including a reduction in agrarian forest areas, impoverishment of soils, disappearance of biodiversity, and food insecurity for the populations have been associated to the deforestation [20]. However, cocoa cultivation that maintains higher proportions of shade trees in a diverse structure (AF cocoa) is progressively being considered as a sustainable land-use practice that meets ecological, biological, and economic objectives [21]. For a long time, AF in cocoa cultivation has also been regarded as environmentally preferable to other forms of cocoa-cropping activities in tropical forest regions [22,23]. By creating favorable microclimatic conditions, AF is viewed as a strategy to sustainably enhance agricultural production, which includes cocoa [24]. It was previously reported that the shade environment produced in AF practices affects the morphology, anatomy, and chemical composition of intercropped forages and therefore may affect forage quality [25]. Our assumption is that the biochemical composition of both pulp and cocoa beans can be affected by the tree shade favored by AF in cocoa cultivation. It is essential to evaluate the balance between the positive and negative effects of AF on the sensory profile of cocoa beans and chocolate, particularly as weather patterns change as a result of climate change. Although Côte d’Ivoire is the world leader producer of raw cocoa beans, up to now, no study really has addressed the issue of cocoa bean sensory and chocolate sensory quality in relation to AF as a relevant cropping system. Moreover, raw cocoa beans sourced from this country are not known for their fine aroma quality [26]. This work investigates the effects of AF as a cropping system on both the volatile compound profiles of cocoa beans and the organoleptic quality of the chocolate produced thereof. To achieve this goal, three major research questions were formulated:

(i) What could be the effect of AF as a cropping system on the sensory profile of cocoa beans?

(ii) Does the cultivation system (AF vs. FS) impact the level of volatile compounds in cocoa beans?

(iii) Does the sensory quality of chocolates made from cocoa beans obtained in the AF or FS systems differ significantly?

## 2. Materials and Methods

### 2.1. Materials

#### 2.1.1. Sites of Carrying out of Research Activities

**Figure 1:**
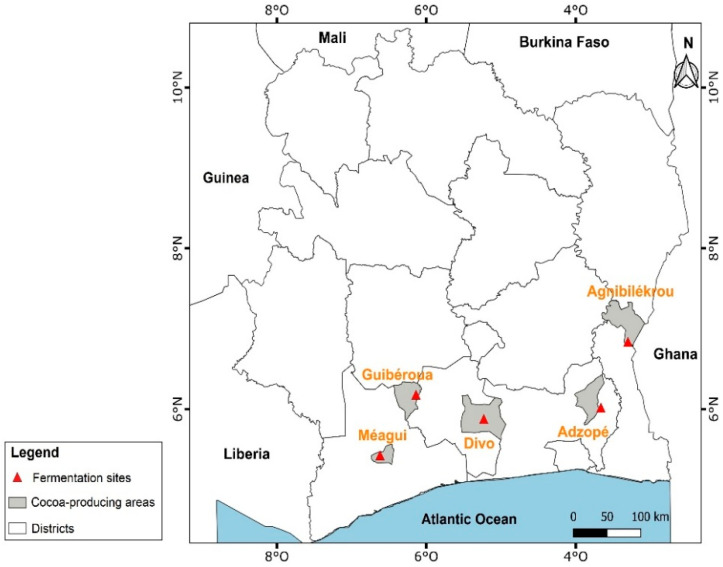
Map of the main cocoa-producing areas in Côte d’Ivoire.

The harvest of ripe cocoa pods and primary cocoa postharvest processing (pod opening, fermentation, and sun-drying) was carried out in 5 main producing areas such as Agnibilekrou (east, geographic coordinates: 7°7′49.012″ N 3°12′11.074″ W), Adzopé (southeast, geographic coordinates: 6°6′25.726″ N 3°51′19.264″ W); Divo (south center, geographic coordinates: 5°49′59.999″ N 5°22′0.001″ W), Guibéroua (west center, geographic coordinates: 6°14′9.805″ N 6°10′16.536″ W), and Méagui (southwest, geographic coordinates: 5°24′00″ N 6°34′00″ O) on both AF and FS system cocoas (Figure 1).

#### 2.1.2. Cocoa Beans Samples

Fresh cocoa beans originating from the mature and full ripe pods of the Ivorian hybrid cultivar, commonly known as “Mercedes” (Amelonado × West African Trinitario), harvested [27] in November 2023 and 2024 from both AF and FS peasant plantation systems were used in this paper. Before the implementation of research activities, partnership agreements between all cocoa producers in each region and the project team were contracted. It is therefore in prior agreement with the owners of the cocoa plantations that we used the different production areas to carry out our research work. So, 5 groups of both AF and FS cocoa bean samples were formed as follows: Group 1 consisted cocoa bean samples from the Méagui region, Group 2 included cocoa bean samples from the Adzopé region, Group 3 comprised cocoa bean samples from the Guibéroua region, Group 4 consisted of cocoa bean samples from the Divo region and Group 5 contained cocoa bean samples from the Agnibilekrou region.

### 2.2. Methods

#### 2.2.1. Fresh Cocoa Beans

The cocoa pods were stored for 3 days before manually opening them with a piece of wooden as a bludgeon, according to the method of Guehi et al. [26]. The fresh cocoa beans were extracted manually without placenta and then sorted to discard the rotten beans. After opening the cocoa pods, approximately 250 g of fresh cocoa beans were taken from each batch of cocoa obtained. These cocoa beans were manually shelled using a scalpel to remove the husk and mucilaginous pulp. The cocoa seeds obtained were rinsed with distilled water before being coarsely ground using a Moulinex grinder (John Gordon^®^, London, UK) [26]. The shreds of the different samples were frozen in liquid nitrogen at −80 °C to be reduced to fine cocoa powder and then stored at −20 °C in a glass vial hermetically closed until analysis of the volatile compounds [27].

#### 2.2.2. Cocoa Beans Fermentation

The cocoa beans fermentation process was simultaneously carried out in wooden boxes in duplicate in all 5 fermentation locations for 6 days regardless of both cropping system and producing areas. We stirred simultaneously the fermenting cocoa beans at days 2 and 4 at all fermentation sites [27]. The fermented cocoa beans were put on the rack daily from 9 a.m. to 17 p.m. for sun drying until reaching 7–8% moisture.

#### 2.2.3. Cocoa Bean Sampling

Three samples of 1000 g of 5-day-fermented cocoa beans were removed from both batches at each fermentation location. All operating workers wore new sterile gloves for the removal of the fermenting cocoa beans from the heap as previously reported by Koné et al. [28]. To ensure moisture removal of 7–8%, every fermented cocoa bean sample was sun-dried on the rack before carrying out chemical analyses [28].

#### 2.2.4. Cocoa Volatile Compounds Analysis

Fifty grams of dry fermented cocoa beans was manually shelled and ground into fine powder (0.5 µm) using a Moulinex grinder (John Gordon^®^, London, UK) and then stored at −20 °C in a glass vial that was hermetically closed [27]. The volatile compounds were extracted from 2.5 g of each cocoa powder sample using the headspace solid-phase micro-extraction technique (HS-SPME) and fibers of 50/30 µm divinylbenzene/carboxene/polydimethylsiloxane (DVB/CAR/PDMS, Supelco, Sigma-Aldrich N.V., Bornem, Belgium) according to the previously described method by Nascimento et al. [29] with n-butanol as standard. Each volatile compound was identified using three criteria: (i) by comparison of the retention index with the database of volatile compounds of the Center for International Cooperation in Agronomic Research for Development (CIRAD) [30], (ii) by matching their mass spectra with those obtained from a commercial database (Wiley275.L, HP product no. G1035 A, Agilent Technologies, Inc., Wilmington, DE, USA), and (iii) whenever possible, the identification was confirmed using pure standards of the components [31]. The formula used to calculate the concentration of each volatile compound was as follows:

$$q_{i}\left(\mu g.g^{-1}\right)=\frac{60\times A_{i}}{A_{but}\times m_{e}}$$

where q_i_ is the relative concentration of the volatile compound; A_i_ is the area of the volatile compound i; A_but_ is the area of 1-butanol (internal standard); 60 is the concentration of 1-butanol in 100 µL of a test sample expressed in µg.g^−1^; m_e_ is the mass of the cocoa powder or the chocolate sample introduced into the vial in g.

The Kovats Retention Index is a dimensionless quantity used to express the relative retention time of a volatile compound in a chromatographic column normalized to the retention time of a reference n-butanol.

#### 2.2.5. Sensory Analysis of Chocolate

Two samples of 2 × 500 g of dry 5-day-fermented cocoa beans were taken from each cocoa bean batch treated at five fermentation locations [27]. Whole cocoa beans were roasted at 125 °C for a duration of 25 min [30]. For the chocolate sensory perception, a trained panel of 12 judges (6 women, 6 men; age, 23–55 years) with experience in dark chocolate evaluation [27] participated in the study. The judges voluntarily agreed to participate in the sensory analysis and authorized the use of their information under a confidentiality agreement proposed by the sensory analysis laboratory of the UMR Qualisud, Montpellier (France). Ethical approval was not required for conducting the sensory evaluation in this study.

They were asked to smell and taste each AF chocolate sample against the FS chocolate as control made from cocoa beans sourced from the same cocoa-producing region. Two training sessions were held in order to calibrate the panel to the selected sensory traits. The performance of the panel was verified using reference chocolates with known sensory profiles and highly perceivable aromatic notes. Sixteen sensory descriptors were simultaneously evaluated using a scale ranging from 0 to 10, and a total score for global quality was assigned to each chocolate sample.

Quantitative descriptive analysis of the chocolate samples was performed according to the methodology reported by Kouassi et al. [27]. Each sample was assessed once. The intensity of 15 attributes and global quality were rated on a continuous line scale from 0 (absent) to 10 (very pronounced). Seven core traits were considered: sweetness, acidity, bitterness, astringency, cacao aroma intensity, cacao taste, and roasted. The complementary attributes included fruitiness, nuttiness, florality, woodiness, spiciness, and vegetal notes.

The sensory analysis was carried out using the “Click and Collect” methodology. This involved a tasting plan for each judge who collected and analyzed chocolate samples. For tasting sessions, FIZZ Software package 3.9.4 (Biosystemes, Saint-Ouen-l′Aumône, France) was used. A tasting plan was developed for each judge, and to randomize the chocolate samples in the sensory analysis, a Latin Square design was employed. The FIZZ software generated three-digit codes and randomized the samples for each judge, allowing the creation of analysis sessions containing four samples. Each judge accessed the FIZZ platform using their credentials, and while conducting the sensory analysis, they recorded the results for each quality trait for every chocolate sample online. Data were collected once all judges completed the sensory analysis of all chocolate samples.

#### 2.2.6. Statistical Analysis

The area of the chromatographic peak of each sensory compound precursor was calculated and then exported using Microsoft Excel 2013. The statistical analyses were carried out with the XLSTAT PLS2 (Addinsoft, New York, NY, USA). Microsoft Excel 2013 (Microsoft Corporation, Redmond, Washington, DC, USA) was used to analyze the data from the sensory perception of each chocolate sample. Principal component analysis (PCA) was used as an unsupervised method to reveal clustering within targeted favor profiles, while partial least squares discriminant analysis (PLS-DA) was employed as a supervised method to identify discriminating volatile compounds across cropping systems and geographical origins of cocoa production [31]. The testing of the equality of variances was performed with the Fischer test with a single factor (*p* < 0.05) in order to indicate the significant differences between volatile compound profiles of cocoa beans and chocolate samples as well as the sensory perception of chocolate produced from tested cocoa as affected by the cropping system or the producing geographical origin [27].

## 3. Results and Discussion

### 3.1. Effect of Agroforestry on Native Volatile Compound Contents of Crude Cocoa Beans

Table 1 indicates that five (5) main classes of volatile compounds including aldehydes, esters, alcohols, ketones, and terpenes were naturally found in crude cocoa beans regardless of both the cropping system and producing geographical origins in Côte d’Ivoire. Similar classes of volatile compounds were found in unfermented cocoa beans by other experiments [32]. The native volatile compound classes in crude cocoa beans could be due to the biological activity of plants that produce a wide spectrum of volatile compounds including aldehydes, alcohols, carboxylic acids, isoprene, and monoterpenes [33]. Among the oxygenated hydrocarbons which are produced by trees, C-1 and C-2 aldehydes, alcohols, and carboxylic acids are of great importance [34]. Cocoa beans from both the Adzopé and Agnibilékrou regions recorded higher concentrations of aldehydes from 102.4 ± 12 to 138.6 ± 7.6 µg.g^−1^ regardless of cropping system. AF cocoa recorded higher aldehydes concentrations than FS cocoa in all producing regions except Divo and Guiberoua. The total concentration of ester class varied from 56 ± 3.5 to 159.8 ± 4.2 µg.g^−1^ regardless of both cocoa cultivation system and cocoa-producing areas. AF cocoas produced in the Méagui region are richest in esters with 159.8 ± 4.2 µg.g^−1^. Furthermore, the AF cocoa bean samples universally contained higher concentrations of esters than the FS cocoas from all of the cocoa-producing regions tested except Agnibilékrou and Divo. C-1 compounds are synthesized during many growth and developmental processes such as seed maturation and the senescence of plant tissues. The production of C-2 compounds, however, seems mainly to be associated with changing environmental conditions, particularly during stress [33]. According to Kreuzwieser et al. [33], acetaldehyde is produced in the leaves of trees if the roots are exposed to anaerobic conditions and produces ethanol through alcoholic fermentation. The production of acetaldehyde and ethanol was not catalyzed by the enzymatic activity of microorganisms including bacteria, fungi, and yeasts. The production of these metabolites could be also ascribed to the fact that when exposed to anoxia or hypoxia, the plant organ could be enriched in the enzymes necessary for fermentation [34]. In addition, aldehyde dehydrogenase enzymes (ALDHs) catalyze the oxidation of a broad range of aliphatic and aldehydes to their corresponding carboxylic acids using NAD^+^ or NADP^+^ as cofactors [35]. The concentration of the alcohols class was the highest, ranging from 173.9 ± 12 to 1134.4 ± 34 µg.g^−1^, while the concentration of the terpenes class was the lowest, between 1.7 ± 0.4 and 12.7 ± 7.2 µg.g^−1^, as previously reported by Yang et al. [32]. AF cocoa beans from the regions of Adzopé, Guibéroua and Méagui recorded a higher concentration in the alcohol family than FS cocoa beans, inversely to cocoa from the regions of Agniblekrou and Divo. Moreover, AF cocoa beans from the Méagui region showed the highest concentration of alcohol with 1134.4 ± 34 µg.g^−1^. The alcohol class concentration varied from 58.6 ± 7 to 504.6 ± 2.7 µg.g^−1^ regardless of the cropping system and producing regions. AF cocoa beans from the areas of Agniblékrou, Guiberoua and Méagui recorded more ketones than FS cocoa. Cocoa beans sourced from the Divo area exhibited similar concentrations in ketones of around 191–195 µg.g^−1^.

**Figure 2:**
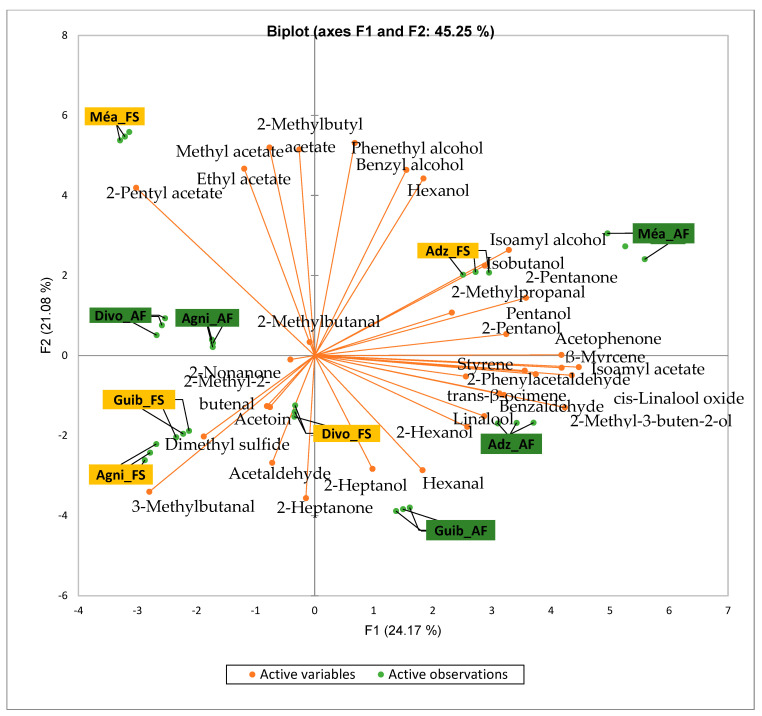
PCA biplot of the sensory profiles of crude cocoa beans according to cropping system. The volatile compounds and cropping systems of the cocoa-producing regions of Côte d’Ivoire are indicated on the labels at the bottom of the figure. Adz = Adzopé region; Agni = Agnibilékrou region; Divo = Divo’s region; Guib = Guibéroua region; Mea = Méagui region.

Figure 2 presents the PCA biplot revealing different groups of unfermented cocoa beans with specific volatile compound profiles produced from the cocoa cultivation system. A total of 34 volatile compounds were found in all of the cocoa bean samples tested. AF has always had a significant influence on sensory profiles. The volatile compound profiles of cocoa beans from AF system were differentiated from those of cocoa beans from FS system throughout all the analyzed production regions except the Agnibilekrou area.

Cocoa bean samples from the Méagui region were mainly characterized by the alcohols such as isoamyl alcohol, isobutanol, pentanol and hexanol. Cocoa bean samples from the Adzopé region recorded various classes of volatile compounds such as benzaldehyde, 2-phenylacetaldehyde, isoamyl acetate, linalool, and β-myrcene. Group 3 consisted of cocoa beans whose sensory profiles recorded 2-heptanol, 2-hexanol, and hexagonal. Volatile compounds such as 2-methyl butanol were detected in the AF cocoa beans from both the Divo and Agnibilekrou areas in Group 4.

The total number of volatile compounds found in our unfermented cocoa bean samples is less abundant than those found in cocoa beans from China, which are 2.5 times higher [36]. The genotype, climatic and pedological factors, soil quality [10], and cropping system may be responsible for these differences. The significant variations observed between the concentrations of some groups of volatile compounds of AF cocoa bean samples from some geographic origins could be due to the variation in AF system types of cocoa cultivation in Côte d’Ivoire. The density of associated trees to the AF system consisted of 48.16 individuals/ha in the west, 22.79 individuals/ha in the central west, and 25.39 individuals/ha in the southwest, as reported by Konan et al. [37]. So, we thought that the shade created by each type of AF system also varied and differentially impacted the soil fertility from cocoa farms to cocoa farms and from producing area to producing area. Indeed, Sauvadet et al. [38] reported that the effects of shade type management are more pronounced on the soil nutrient availability through changes in the soil food web structure than on the direct organic chemical composition of crops, highlighting the importance of choosing shade tree species in an AF system. Moreover, the AF system in the central west and southwest is dominated by trees taller than 8 m with a high density of associated perennial crops in the central west, while the AF systems in the southwest are characterized by plots averaging 30 years in age [37]. To conclude, AF in cocoa cultivation influenced the native volatile compounds of Ivorian cocoa beans. But the significant variation observed in the quality of volatile compound profiles depends on the producing geographical origins due to the type of AF and probably climatic factors as well. Our study is relevant and could be continued by implementing the same AF system regarding the density and height of trees, the soil quality, the local climatic factors of each area, and the age of the studied cocoa trees, which seems to impact the sensory profile of the crude cocoa beans [10].

### 3.2. Effect of Agroforestry on Sensory Compound Contents of Dry Fermented Cocoa Beans

Seven (7) main classes of sensory compounds, including aldehydes, esters, alcohols, ketones, acids, pyrazines, and terpenes, were detected in the dry fermented cocoa bean samples regardless of both the cropping system and producing regions in Côte d’Ivoire (Table 2). In the dry fermented cocoa beans, the concentrations of each sensory compound class were significantly increased. Also, two new classes such as acids and pyrazines occurred in comparison to unfermented cocoa beans regardless of both the cropping system and the cocoa-producing region. Fermentation led to the development of specific cocoa volatile compounds via the degradation of proteins and formation of volatile compounds, such as pyrazines, which were described as one of the few classes of compounds with desirable sensory properties [36]. AF dry fermented cocoa beans recorded higher concentrations of aldehydes than FS cocoa beans in the Guibéroua and Méagui regions, while lower concentrations were recorded in cocoa beans sourced from the Agnibilékrou and Divo regions. Cocoa bean samples from the Adzopé region recorded the highest concentrations of aldehydes (456.08 ± 77.6 µg.g^−1^), while those from the Guibéroua region recorded the highest concentration of esters with about 117. 68 ± 9.7 µg.g^−1^. Alcohol concentrations of dry fermented cocoa beans varied from 83.31 ± 1.2 to 385.67 ± 259.1 µg.g^−1^. The concentration of ketones was relatively constant; the approximate values ranged between 40.55 ± 12.76 and 154.46 ± 4.9 µg.g^−1^ regardless of both the cropping system and the cocoa-producing area. All tested dry fermented cocoa bean samples contained pyrazines. However, the highest concentration of pyrazines was recorded in dry fermented cocoa beans sourced from the Divo region (70.61 ± 28.51 µg.g^−1^).

**Figure 3:**
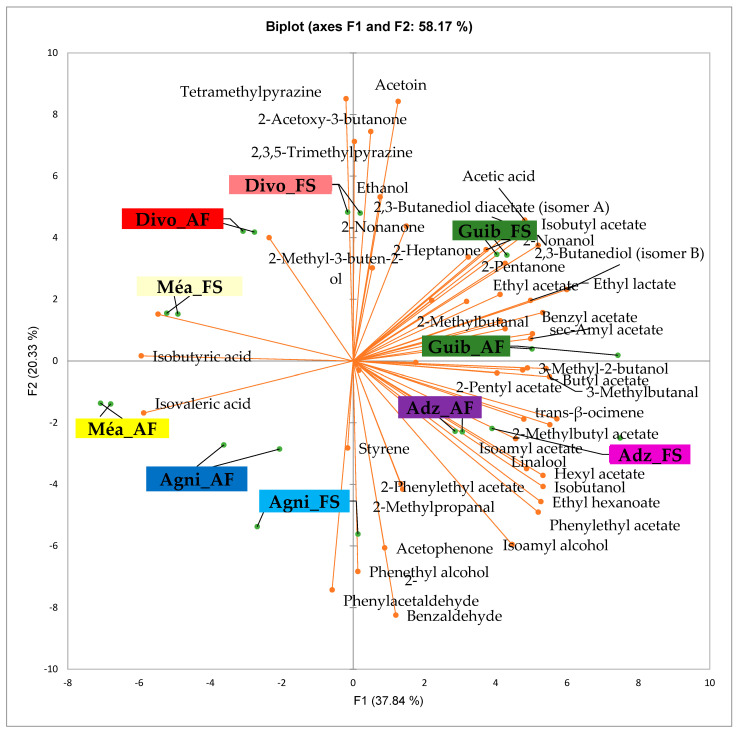
PCA biplot of the sensory profiles of dry fermented cocoa beans according to cropping system. The volatile compounds and cropping systems of the cocoa-producing regions in Côte d’Ivoire are indicated on the labels at the bottom of the figure. Adz = Adzopé region; Agni = Agnibilékrou region; Divo= Divo region; Guib = Guibéroua region; Mea = Méagui region. (AF = agroforestry system; FS = full sun system).

Figure 3 presents the PCA biplot, which reveals different groups of dry fermented cocoa beans regarding their sensory compound profiles according to the cocoa cultivation system between all production areas tested. A total of 49 volatile compounds were found in all of the dry fermented cocoa bean samples tested. The volatile compound profiles of cocoa beans from the AF system were not differentiated from those of FS cocoa beans, while the geographical origins did show a significant influence.

Group 1 consisted of both AF and FS cocoa bean samples from the Méagui region. The volatile compound profiles contained primarily isoamyl alcohol, phenylethyl acetate, ethyl acetate, ethyl hexanoate, linalool, hexyl acetate, methylethyl acetate, methyl butanol, butyl acetate, 2 acetate, 2-methylpropanal, and ethyl octanoate. Group 2 included both AF and FS cocoa beans from the Adzopé area. Its volatile compound profile contained more sensory compounds, including isoamyl alcohol, phenylethyl acetate, ethyl hexanoate, linalool, methyl, ethyl acetate, methyl butanol, butyl acetate, 2-pentyl acetate, and 2-methyl propane. Group 3 included both AF and FS cocoa bean samples sourced from the Guibéroua region. These cocoa bean samples exhibited volatile compound profiles consisting of various classes such as benzylacetate, sec amyl acetate, 2 heptanol, 2-methyl butanol, 2,3-butanol acetate, 2,3 butanediol, ethylacetate, 2,2-butanediol acetate, acetic acid, isobutyl acetate, 2-nonanol, and 2-heptanone. Group 5 consisted of cocoa bean samples from the Agnibilékrou region which were characterized by no specific volatile compounds, whereas Group 4 consisted of cocoa bean samples from the Divo region and contained acetoin, 2-acetoxy-3-butanone, 2,3,5-trimethyl pyrazine, ethanol.

**Figure 4:**
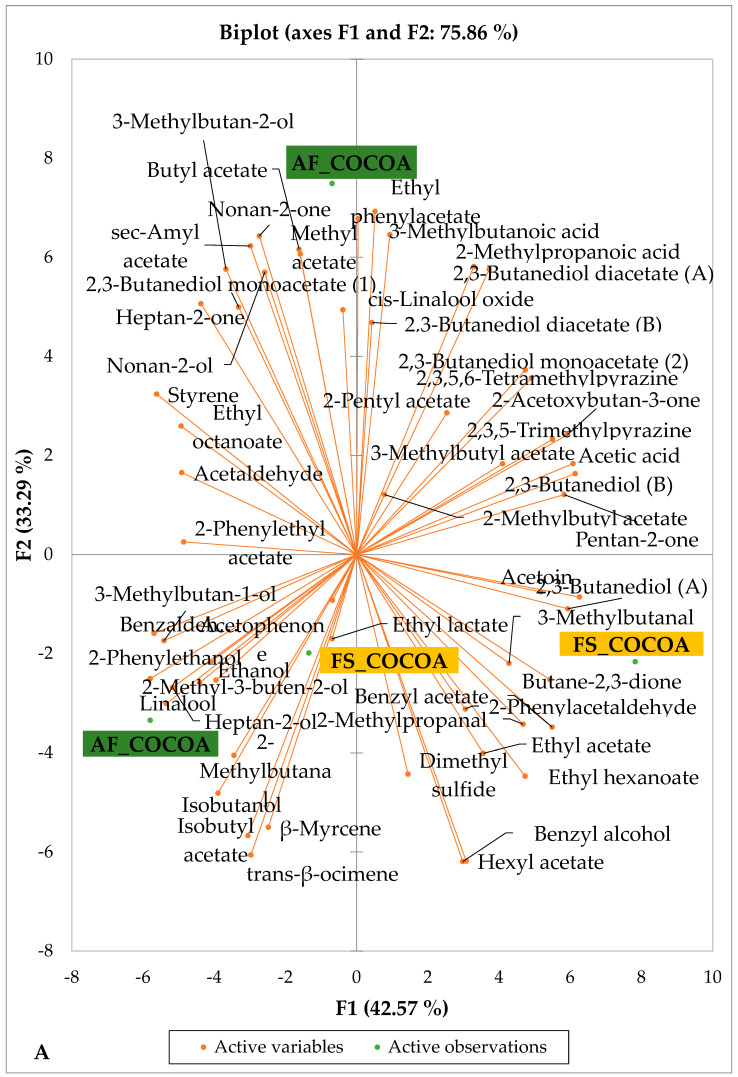

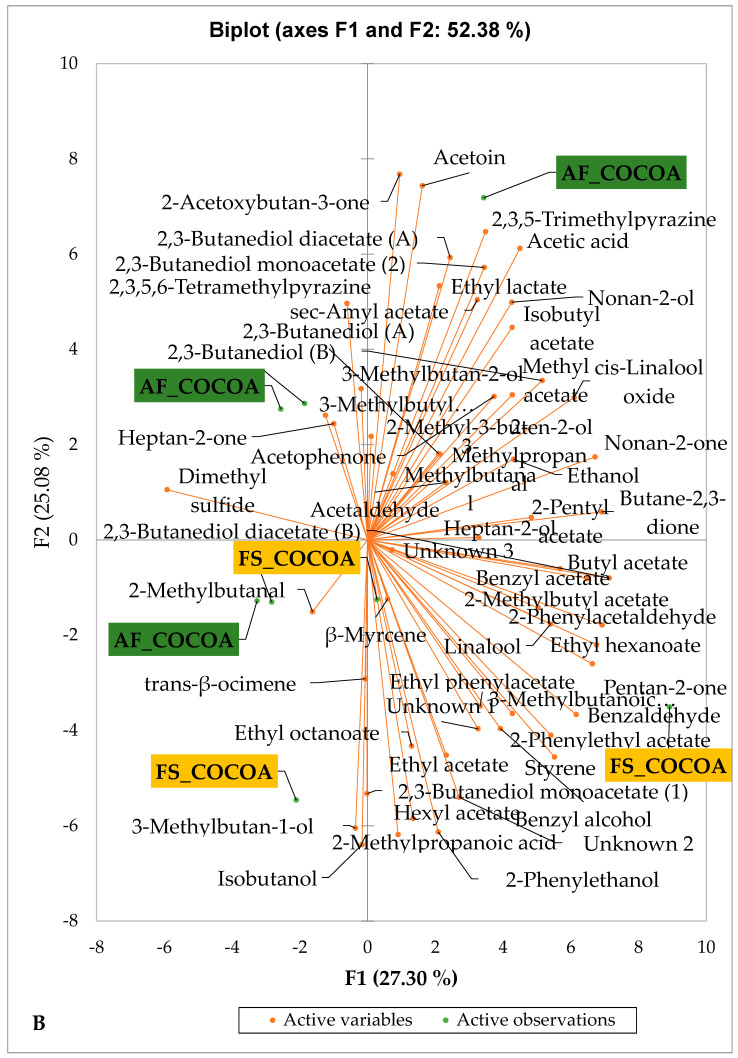

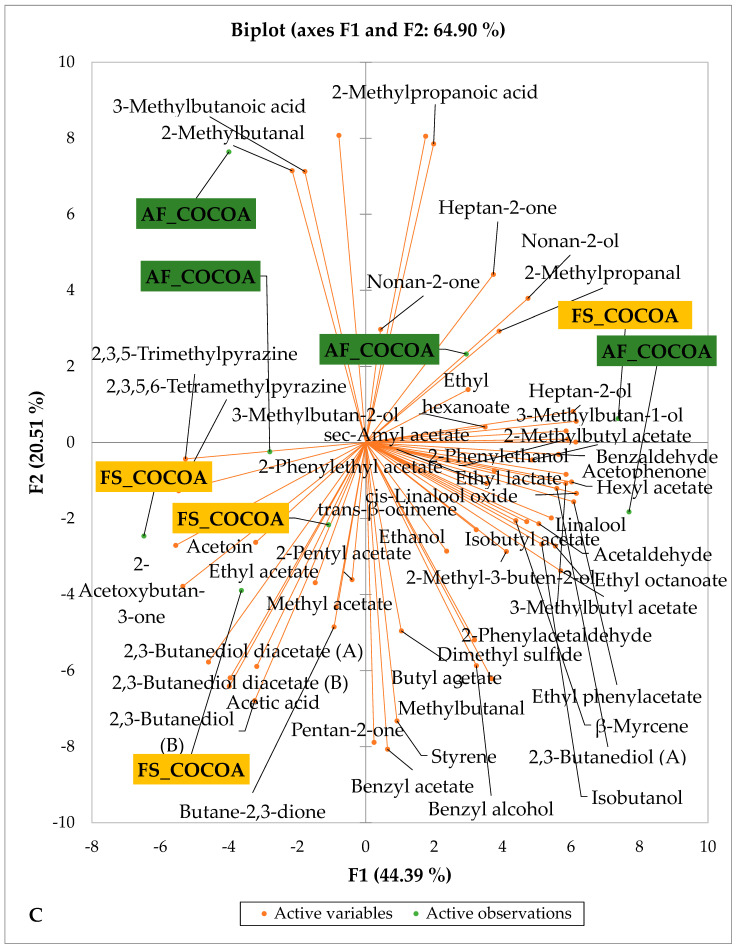

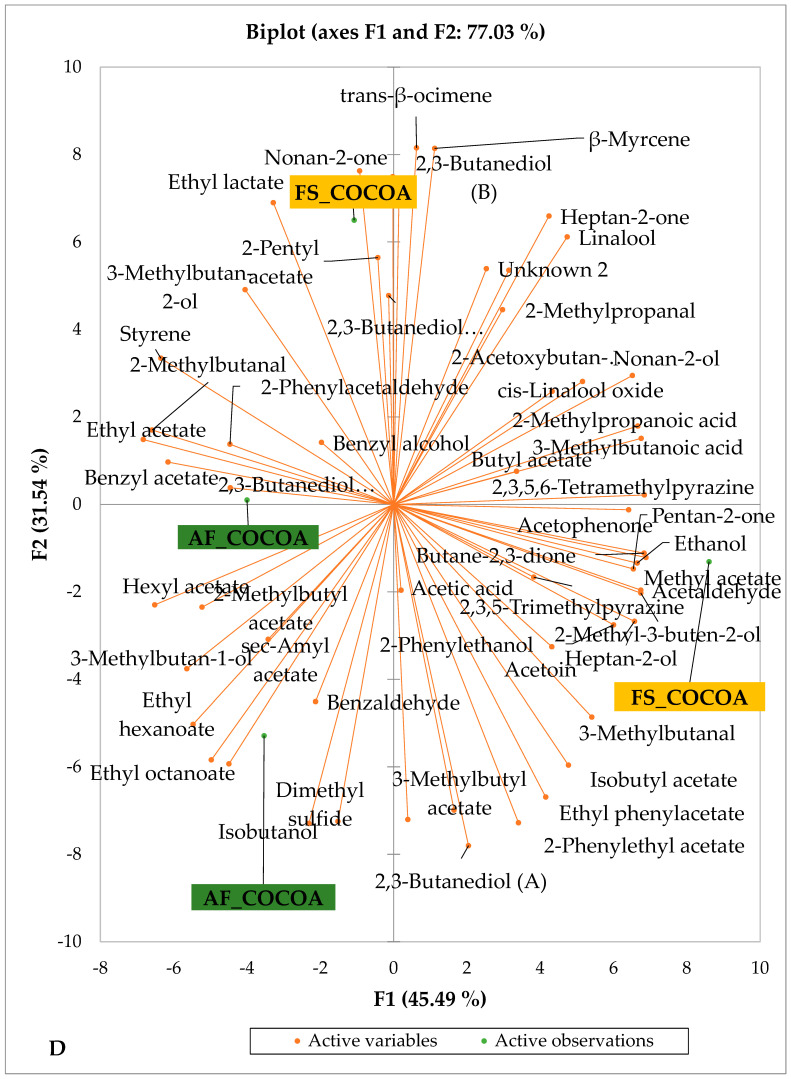

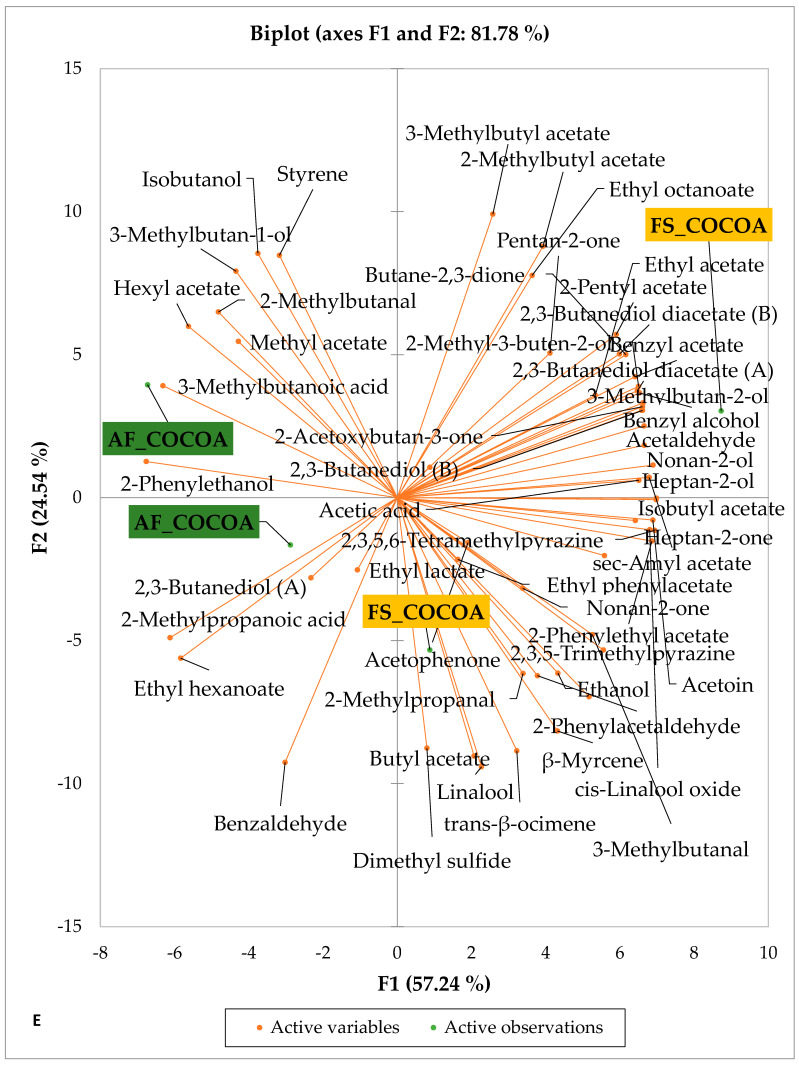
PCA biplot of the sensory profiles of dry fermented cocoa beans according to cropping system. The volatile compounds and cropping system of the cocoa-producing regions in Côte d’Ivoire are indicated on the labels at the bottom of the figure. (AF = agroforestry system; FS = full sun system). (**A**) Adzopé region; (**B**) Agnibilékrou region; (**C**) Divo region; (**D**) Guibéroua region; (**E**) Méagui region.

Figure 4 presents the PCA biplot, which reveals the sensory compound profiles of different groups of dry fermented cocoa beans according to the cocoa cultivation system within each cocoa-producing area tested. AF had a significant effect on the sensory profiles of dry fermented cocoa beans from only two cocoa-producing areas, including the Guiberoua and Méagui regions. The volatile compound profiles of cocoa beans from the AF and FS systems sourced from the Adzopé, Agnibilékrou, and Divo regions were similar.

Our results regarding the presence of seven classes of sensory compounds in dry fermented cocoa bean samples are in agreement with those of several previous works [7,27,30,36,39,40,41,42]. The appearance of new classes, including acids and pyrazines, in the beans could be due to the microbial activity comprising yeast and acetic acid bacteria during cocoa fermentation, as previously reported by Fang et al. [35]. Indeed, several volatile compounds not found in fresh beans gradually produced after fermentation and appeared in dry fermented cocoa beans. Our results regarding the detection of a pyrazines class in all of our tested dry fermented cocoa bean samples regardless of both cropping system and production area are in agreement with those reported by several works [39,43,44]. However, several other researchers reported that pyrazines are formed in cocoa beans only during roasting [45,46]. Yet, recent work has highlighted that the pyrazines are formed in food via both thermal treatment and fermentation [47]. Furthermore, for a long time, it was reported that pyrazines are formed in cocoa beans during fermentation due to the enzymatic activities produced by Bacillus sp. [43,48], but their concentration was increased by thermal treatment notably during the roasting [49]. The acidification of cocoa beans could be ascribed to the production of a high amount of acetic acid by acetic acid bacteria during fermentation, which induces various biochemical reactions and pathways leading to the development of other various cocoa sensory compounds [36]. The acidification process of cocoa beans is largely influenced by the acetic acid content, which closely correlates with the pH level of the beans. [50]. In addition, the aldehydes, esters, and acids classes recorded higher concentrations, while the pyrazines and terpenes families presented lower concentrations, as previously reported [36,39]. It is possible that the increased acid content is responsible for the creation of esters and higher alcohols [51]. The highest concentration of aldehydes in some of the AF and FS dry fermented cocoa beans from the Guiberoua and Méagui regions could be due to the activity of *Galactomyces geotrichum*, which is a aldehydes-producing yeast species currently involved in cocoa fermentation in Côte d’Ivoire [28]. The high concentrations of alcohols in AF dry fermented cocoa beans could be due to the wide amounts of fermentable sugars of cocoa pulp [52] and the involvement of high alcohols producing yeast species including *Candida tropicalis*, *Wikehamoyces anomalus*, *Pichia kudriazevii*, *Saccharomyces cerevisae*, *Pichia galieformis* [28]. Ethanol and acetic acid are the primary molecules generated when cocoa pulp substrates, such as sugars, citric acid, and polyphenols, are metabolized [53]. Indeed, the AF system favors a higher amount of substrates in AF cocoa pulp than the FS cocoa does via the soil fertilization, as previously reported [38]. To conclude, although AF influences the native sensory compounds of cocoa beans, it does not influence the sensory profiles of dry fermented beans, which is probably due to the activity of the yeast involved in cocoa fermentation. However, regarding each cocoa-producing area, we observed that AF influenced the volatile compound profiles of dry fermented cocoa beans from the Guibéroua and Méagui areas in the central west of Côte d’Ivoire. These results could be ascribed to the type of AF system in this region, which is dominated by trees taller than 8 m with a high density of associated perennial crops [37], the climatic factors, the soil quality [10], and probably the community of yeast species involved in the fermentation process [27].

### 3.3. Effects of Agroforestry and Producing Regions on the Desired Volatile Compound Profiles of Cocoa Beans 

Table 3 shows the concentration of several positive sensory compounds of dry fermented cocoa beans regarding both the AF system and the cocoa-producing regions. A total of 15 useful sensory compounds were found in all of the dry fermented cocoa beans analyzed regardless of the cropping system. The detected desirable sensory attributes belong to various classes such as esters (3-methylbutyl acetate, 2-methylbutyl acetate, benzyl acetate), aldehydes (2-methylpropanal, 2-methylbutanal, 3-methylbutanal), alcohols (benzyl alcohol, 2-phenylethanol, linalool), ketones (acetoin, 2-acetoxybutan-3-one, acetophenone), and pyrazines such as 2,3,5-trimethylpyrazine and tetramethylpyrazine. The fermentation process significantly influences cocoa’s sensory attributes and aroma [54]. Several desirable volatile compounds were detected at the highest concentrations in AF dry fermented cocoa beans from the Guiberoua region. Indeed, 2-methyl butyl acetate, 3-methyl butyl acetate and benzyl acetate were found at 84.2 ± 3.7, 76.3 ± 11.8, and 3.4 ± 2.3 µg.g^−1^ concentrations respectively. Regarding aldehydes, AF dry fermented cocoa beans recorded 65.32 ± 3.32 µg.g^−1^ of methyl butanol, while FS cocoa beans recorded 26.3 ± 9.7 µg.g^−1^. In the class of alcohols, 2-phenylethanol was found at the highest concentrations of 75.4 ± 54.3 and 22.7 ± 8.2 kg.g^−1^ µg.g^−1^ in AF and FS cocoa bean samples, respectively. Among pyrazines, tetramethyl pyrazine was found at the highest concentration in dry fermentation produced in the Guiberoua area. The concentration of specific acetate esters including ethyl acetate and phenyl ethyl acetate can be favored by fermentation techniques like turning the beans [55]. The richness of Gubéroua cocoa in various relevant volatile compounds may be due to the sensory attributes produced by yeast species such as *Saccharomyces cerevisiae*, *Pichia kudriazevii* and *Hanseniaspora opuntia* in the fermentation of cocoa carried out in this region [28,56]. Furthermore, Crafack et al. [54] have previously demonstrated that several sensory compounds are produced primarily by yeast and bacteria during the fermentation process, which converts amino acids and higher alcohols into various volatile compound precursors. According to Ho et al. [46] and Ziegleder [57], the volatile compound profile of chocolate is enhanced by significant increases in esters concentration during spontaneous fermentation. The distinct floral and fruity notes that distinguish premium chocolate from bulk varieties are due to other key compounds like linalool (found in fine cocoa varieties) and phenylethyl acetate [12]. Linalool is the essential volatile compound that forms during cocoa fermentation, as Ziegleder [58] has reported for a considerable amount of time. Our finding that tetramethyl pyrazine is the main compound of the pralines class was in accordance with those of previous studies [59]. Although several works reported that the pyrazine group is more common in well-fermented roasted beans, these metabolic products of Bacillus species (*B. subtilis* or *B. megaterium*) are formed at the end of cocoa fermentation [40,41,60,61]. Each sensory compound could contribute to the final volatile compound profile of dry fermented cocoa beans. For example, pyrazine compounds have cocoa, chocolate, walnut, popcorn, coconut, candies, and fruity notes [42]. However, certain volatile compounds were more concentrated in AF cocoa beans than those of FS from the Guibéroua region. The impact AF on the formation of crucial volatile compounds in dry-fermented cocoa beans has yet to be shown.

### 3.4. Effects of Agroforestry on the Volatile Compound Profiles of Chocolates Within Each Cocoa-Producing Area

**Figure 5:**
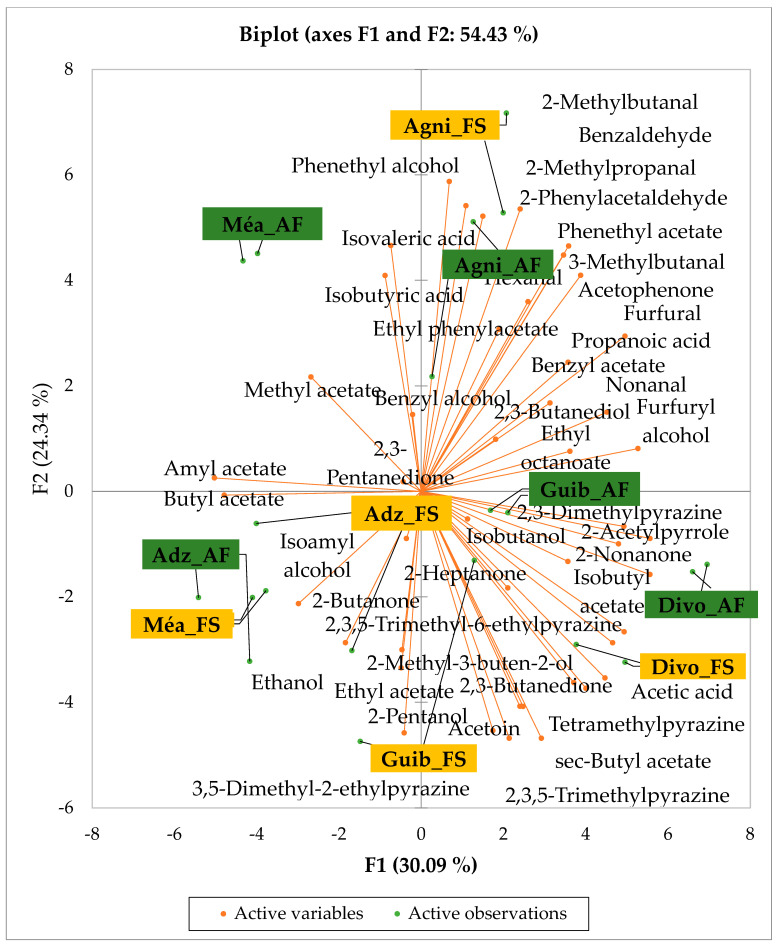
PCA biplot of sensory compounds in chocolates produced from dry fermented cocoa beans produced according to cropping system and producing region. The volatile compounds and cropping system of the cocoa-producing regions in Côte d’Ivoire are indicated on the labels at the bottom of the figure. Adz = Adzopé region; Agni = Agnibilékrou region; Divo = Divo region; Guib = Guibéroua region; Mea = Méagui region. (AF = agroforestry system; FS = full sun system).

**Figure 6:**
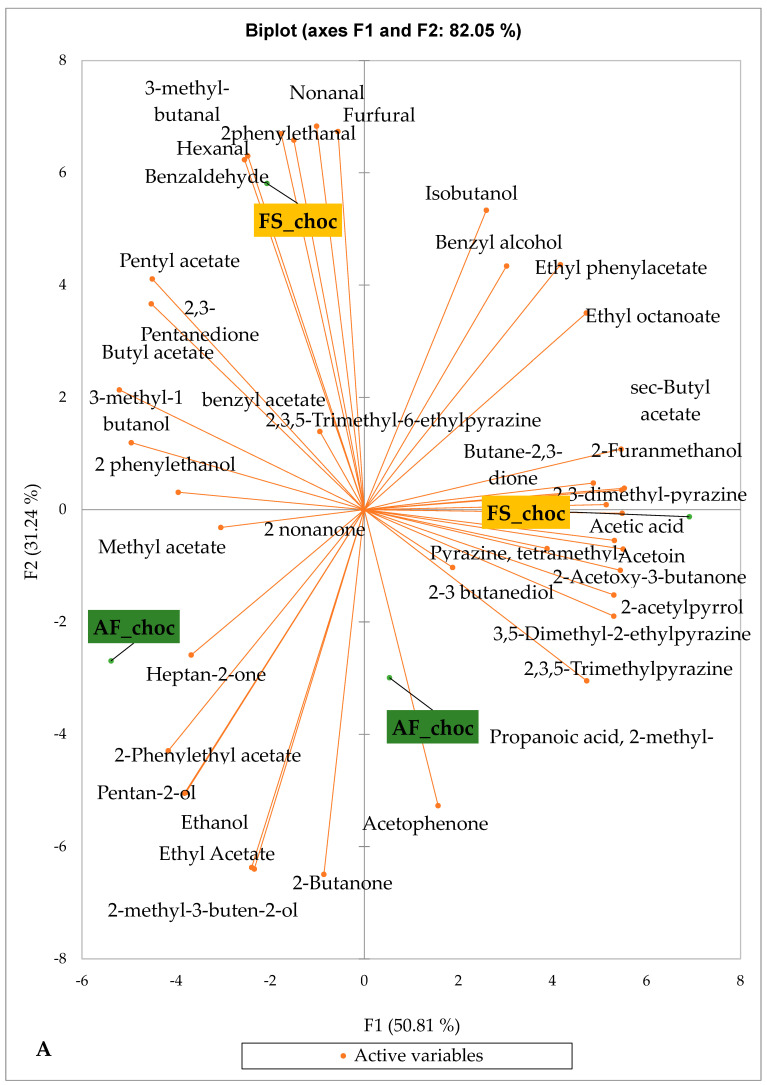

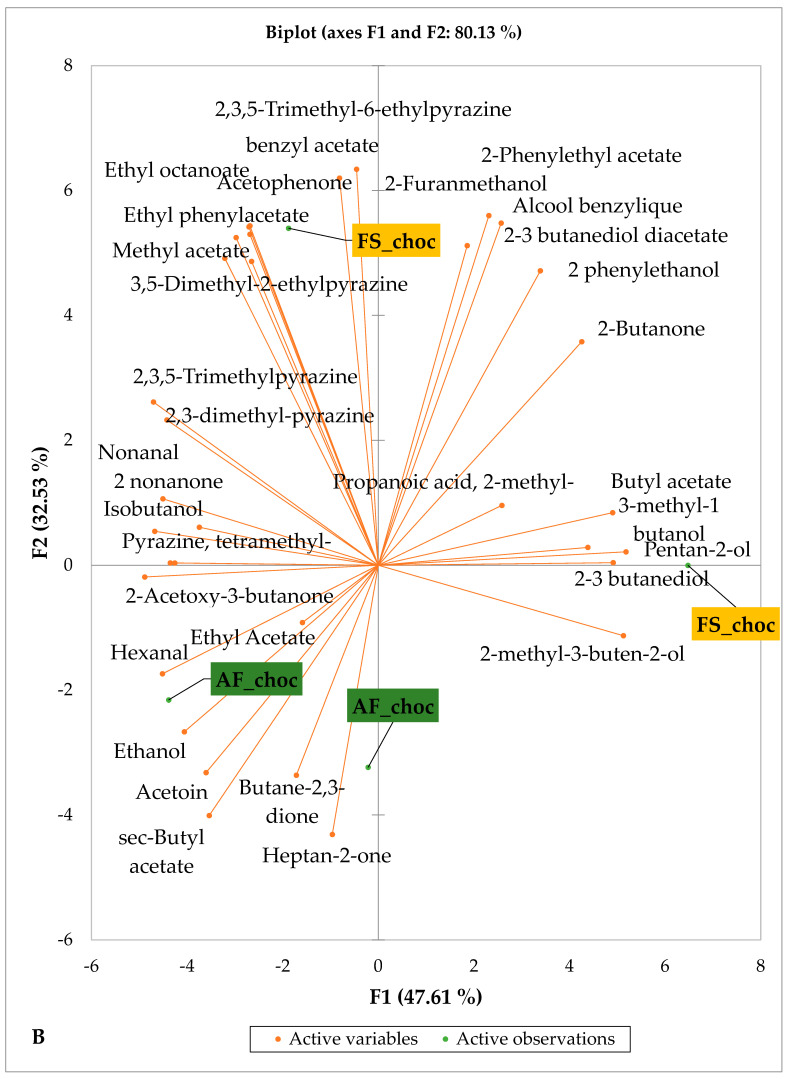

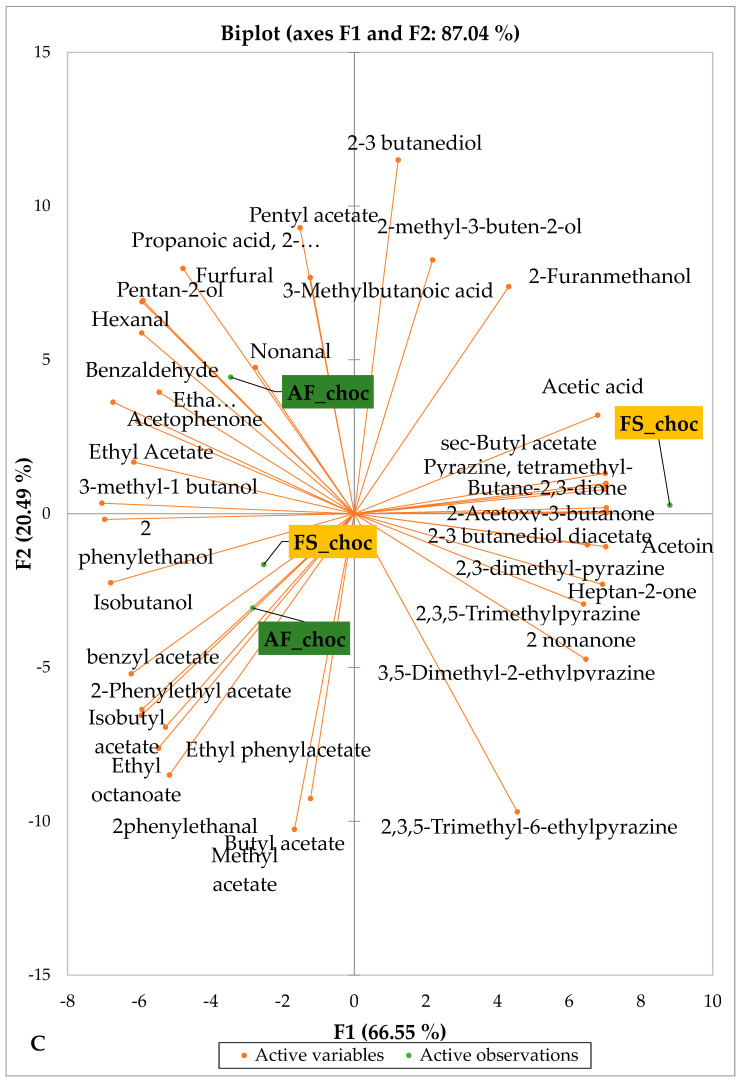

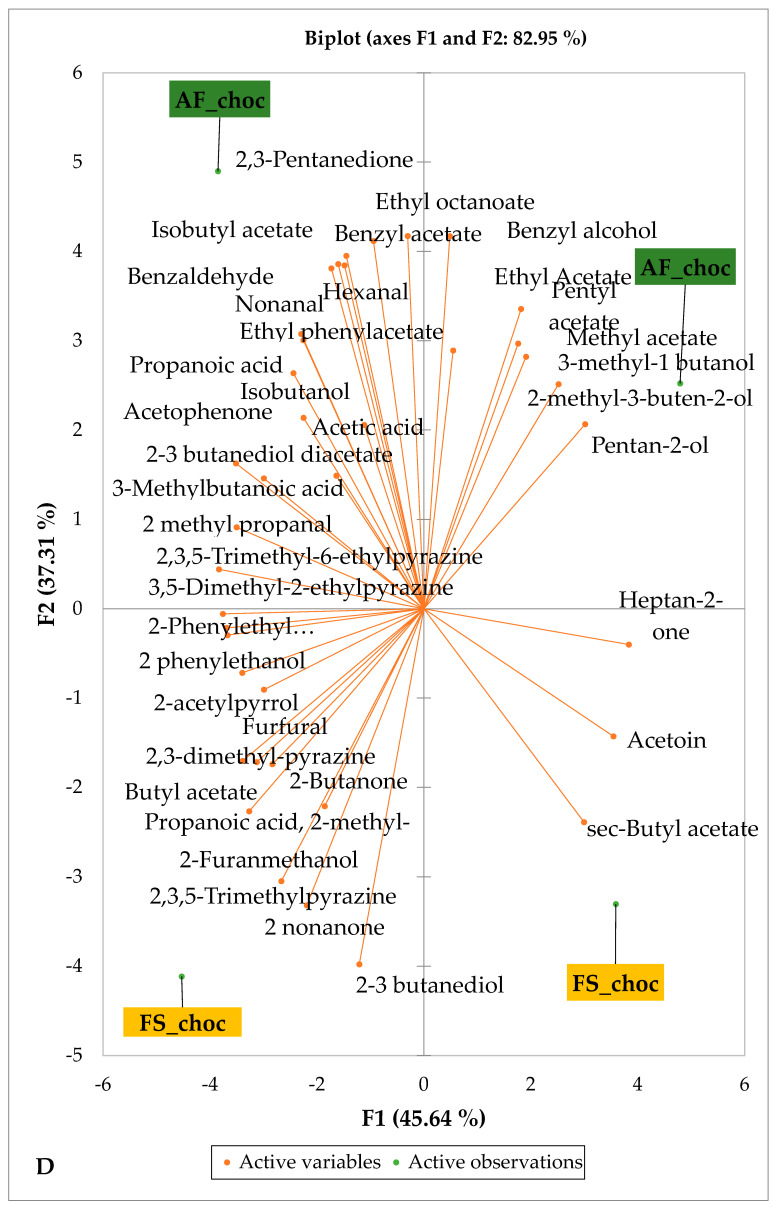

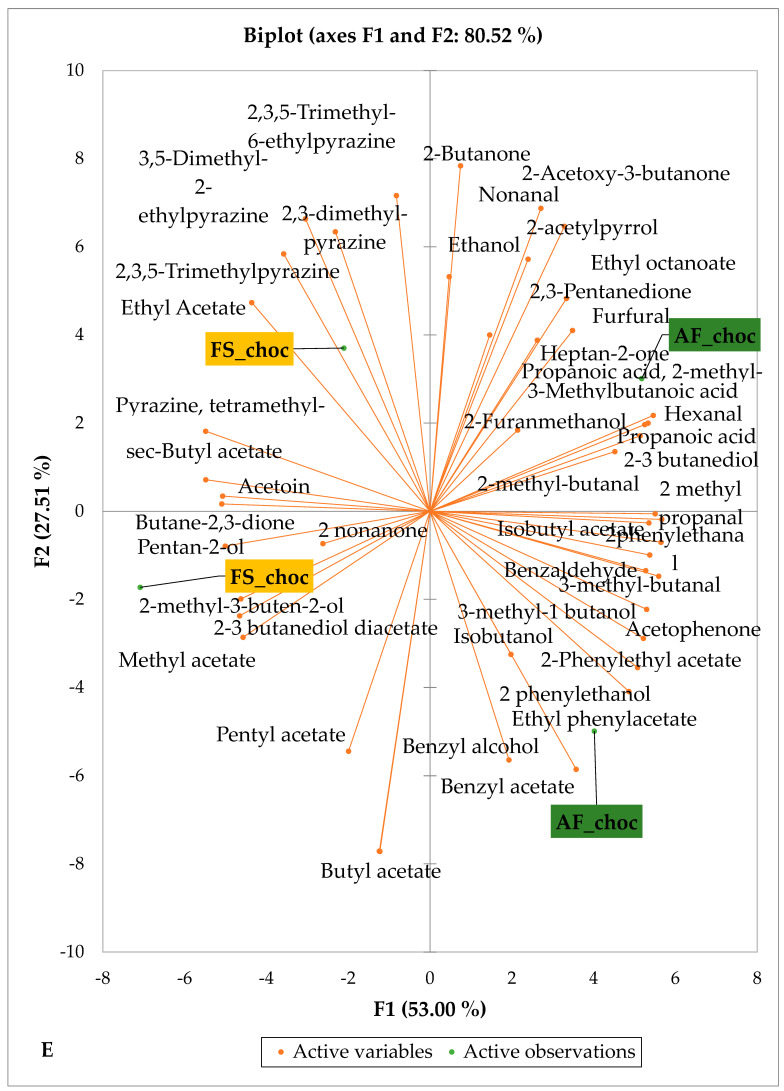
PCA biplot of sensory compounds in chocolates produced from dry fermented cocoa beans according to the cropping system within producing regions. The sensory compounds and cropping systems of the cocoa-producing regions in Côte d’Ivoire are indicated on the labels at the bottom of the figure. (AF = agroforestry system; FS = full sun system). (**A**) Adzopé region; (**B**) Agnibilékrou region; (**C**) Divo region; (**D**) Guibéroua region; (**E**) Méagui region.

Figure 5 and Figure 6 indicate the PCA biplot, which reveals the effects of AF on the sensory profiles of chocolate both between and within the production areas tested, respectively. The comparison between cocoa-producing areas revealed that the sensory profile of chocolate produced from AF cocoa beans was only distinctive from that of chocolate from FS cocoa beans in the Méagui region. However, our findings about each cocoa-producing area showed that the sensory compound profiles of chocolate derived from AF cocoa beans sourced from the areas of Agnibilékrou (Figure 6B) and Méagui (Figure 6E) were distinctive from those of the chocolate samples derived from FS cocoas within the corresponding areas. AF has shown no significant effect on the sensory profiles of the chocolates issued from other cocoa-producing areas: the Adzopé (Figure 6A), Divo (Figure 6C), and Guiberoua (Figure 6D) regions. The sensory profiles of AF chocolate from the Agnibilékrou region included hexanal, ethyl acetate, ethanol, sec butyl acetate, acetoin and butane-2,3-dione, while those from FS dry fermented cocoa beans included 2,3-butanediol, butyl acetate, 3 methyl butanol, pentan-2-ol, benzyl acetate, ethyl octonate, ethyl phenyl acetate, 3,5 dimethyl-2-ethyl pyrazine, acetophenone, 2-phenyl acetate, and 2,3,5-trimethyl-6-ethyl pyrazine. In the Méagui region, the chocolate derived from AF cocoa beans contained more volatile compounds (23) than those derived from FS cocoa. The sensory profile of this AF chocolate included several key volatile compounds: benzyl alcohol, iso-butanol, benzyl acetate, ethyl phenylacetate, 2-phenyl butanol, benzaldehyde, 2-phenyl ethanol, isobutyl acetate, and 3-methyl butanoic acid. Recall that the sensory profiles of AF dry fermented cocoa beans from Méagui and Agnibilekrou consist of isobutyric and isomeric acids. Isobutyric acid is present in cocoa beans, being formed during the fermentation and drying processes from the decomposition of sugars and other compounds [62]. Although it contributes to the overall sensory profile of the beans, its concentration may vary based on factors such as fermentation time, bean variety, and subsequent processing like roasting [63]. However, isovaleric acid is present in cocoa beans, where it is a volatile organic acid that develops during fermentation. It is produced by microorganisms and contributes to the acidification of the beans, though it is considered an undesirable compound when present in high concentrations, as it can produce a “rancid” smell. The sensory quality of chocolate broadly depends on the volatile compound profile resulting from microbial metabolism during cocoa bean fermentation [64]. The discrimination of the sensory profiles of chocolates derived from AF cocoa beans produced in the Agnibilékrou and Méagui regions could be due to the presence of either one, the other, or even both isovaleric and isobutyric acids.

### 3.5. Effects of Agroforestry on the Organoleptic Quality of Chocolate Samples

**Figure 7:**
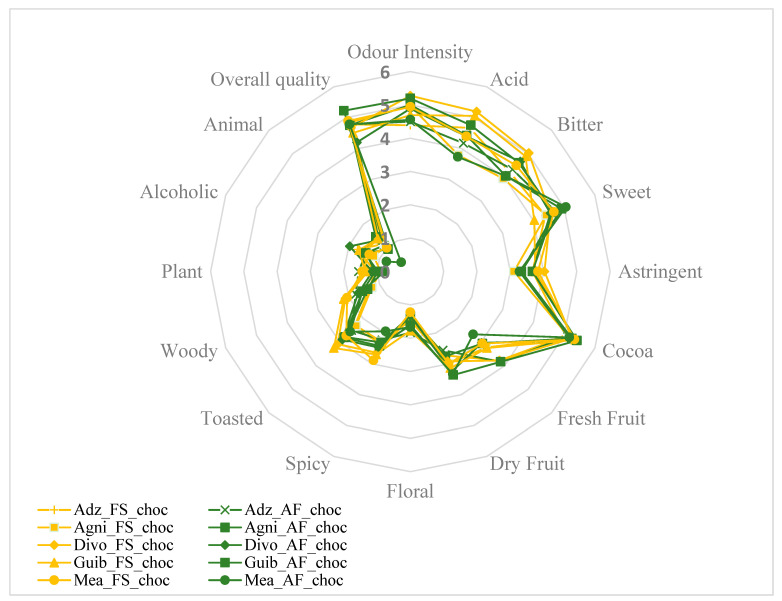
Effects of agroforestry on the sensory attributes of chocolate samples made from cocoa beans between different cocoa-producing areas in Côte d’Ivoire. Adz_FS_choc: Chocolate derived from FS cocoa beans from Adzopé region; Adz_AF_choc: Chocolate derived from AF cocoa beans from Adzopé région; Agni_FS_choc: Chocolate derived from FS cocoa beans from Agnibilékrou region; Agni_AF_choc: Chocolate derived from AF cocoa beans from Agnibilékrou region; Divo_FS_choc: Chocolate derived from FS cocoa beans from Divo region; Divo_AF_choc: Chocolate derived from AF cocoa beans from Divo region; Guib_FS_choc: Chocolate derived from FS cocoa beans from Guibéroua region; Guib_AF_choc: Chocolate derived from AF cocoa beans from Guibéroua region; Méa_FS_choc: Chocolate derived from FS cocoa beans from Méagui region; Méa_AF_choc: Chocolate derived from AF cocoa beans from Méagui region.

**Figure 8:**
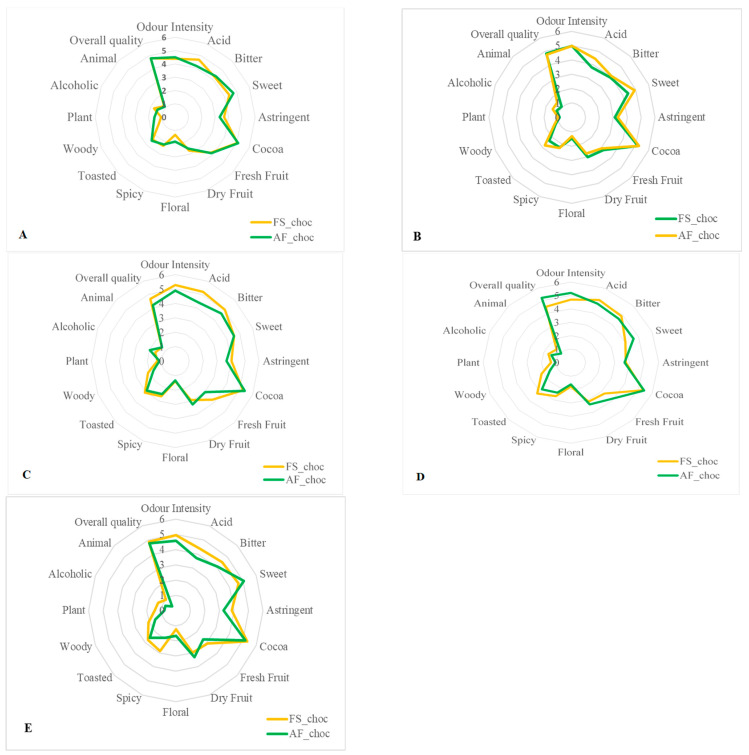
Effects of agroforestry on the sensory attributes of chocolate samples made from cocoa beans produced within each tested cocoa-producing area in Côte d’Ivoire. (**A**) Adzopé region, (**B**) Agnibilekrou region, (**C**) Divo region, (**D**) Guibéroua region, (**E**) Méagui region.

Sixteen descriptive attributes, namely intensity of odor, acid, bitter, sweet, astringent, cocoa aroma, fresh fruit, dried fruit, floral, spicy, toasted, woody, plant, alcoholic aroma, animal, and global quality, were evaluated. The average of the score attributed for each sensory property to the finished chocolates from each fermented cocoa sample was then calculated. Figure 7 and Figure 8A,B show that all end-chocolate samples present the same score for almost all descriptive attributes regardless both of cropping system and geographical origin. Neither AF nor the cocoa production area influenced the sensory perception of the manufactured chocolates.

## 4. Conclusions

This paper investigated the effect of AF on the sensory profiles of cocoa beans and the chocolate’s sensory perception. The AF system significantly influenced the native sensory profiles of raw cocoa beans regardless of the geographical origin. After transformation (fermentation and roasting), the difference in sensory compound profiles is not as significant as it was in fresh cocoa beans because of the yeasts and thermal treatments such as roasting. Furthermore, the AF system influenced the volatile compound profile of both dry fermented cocoa beans and end-chocolate as a function of the cocoa-producing region, but it showed no effect on the organoleptic quality of the end-chocolate. The AF system can therefore be promoted in cocoa cultivation around the world in order to contribute to the conservation and restoration of the biosphere and ensure the sustainability of cocoa. The outcome of this work will be to evaluate the effect of both soil quality and AF featuring the same density and the same species of trees of even height on the organoleptic qualities of chocolate.

## Figures and Tables

**Table 1:** Changes in total concentration of each main class of sensory compounds of crude cocoa beans as a function of the cropping system in different cocoa-producing areas of Côte d’Ivoire.

Chemical Families	Sensory Compounds Content (µg.g^−1^) per Class Found in Crude Cocoa Beans According to Ivorian Cocoa Producing Areas									
	Adzopé		Agnibilékrou		Divo		Guibéroua		Méagui	
	Cropping Systems									
	*AF System*	*FS System*	*AF System*	*FS System*	*AF System*	*FS System*	*AF System*	*FS System*	*AF System*	*FS System*
Aldehydes	138.6 ± 7.6 ^a^	118.8 ± 2.4 ^a^	111.7 ± 4.6 ^ab^	102.4 ± 12.8 ^a^	73.7 ± 0.9 ^c^	116.5 ± 2.7 ^a^	107.6 ± 23.5 ^b^	116.2 ± 41.1 ^a^	95.7 ± 1.2 ^bc^	92.7 ± 1 ^a^
Esters	85.4 ± 2^b^	59.5 ± 2.5 ^a^	56 ± 3.5 ^c^	70.1 ± 25.7 ^a^	56.6 ± 0.3b ^c^	73.2 ± 2.8 ^a^	65 ± 22.1b ^c^	56.2 ± 25.1 ^a^	159.8 ± 4.2 ^a^	71.7 ± 1.4 ^a^
Alcohols	590.9 ± 27.4 ^b^	391.6 ± 10 ^a^	330.6 ± 110.8 ^c^	448.6 ± 223.7 ^a^	341.5 ± 9.1 ^c^	432.2 ± 0.1 ^a^	432 ± 69.2 ^c^	362 ± 186.1 ^a^	1134.4 ± 34 ^a^	173.9 ± 12 ^a^
Ketones	103.4 ± 6 ^c^	151.7 ± 0.9 ^a^	108.7 ± 38.7 ^c^	85.8 ± 62 ^a^	191.4 ± 32.4 ^b^	195.1 ± 1.1 ^a^	153.3 ± 5.7 ^b^	106.7 ± 91.3 ^a^	504.6 ± 2.7 ^a^	58.6 ± 7 ^a^
Terpenes	8.5 ± 0 ^ab^	11 ± 0.7 ^a^	3.9 ± 0.5 ^b^	2.7 ± 0.3 ^c^	1.7 ± 0.4 ^b^	4.3 ± 0.3 ^b^	12.7 ± 7.2 ^a^	3.2 ± 0.5 ^c^	8.2 ± 0.1 ^ab^	2.9 ± 0.1 ^c^
Others	7.7 ± 0 ^a^	3.2 ± 0.1 ^c^	7.7 ± 8.1 ^a^	4.6 ± 3 ^bc^	5.9 ± 0.4 ^a^	8 ± 0.2 ^ab^	5.4 ± 1 ^a^	2.3 ± 1.2 ^c^	6.4 ± 0.1 ^a^	10.3 ± 0.5 ^a^

AF = agroforestry, FS = full sun. The superscript letters indicate significant differences between the concentrations of some desirable volatile compound classes of cocoa bean samples from AF systems and cocoa bean samples based on cocoa-producing region according to the Fischer test at *p* < 0.05.

**Table 2:** Changes in total concentration of each class of volatile compounds (µg.g^−1^) of AF dry fermented cocoa beans from different cocoa-producing region of Côte d’Ivoire.

Classes of Volatile Compounds	Adzopé	Agnibilékrou	Divo	Guibéroua	Méagui
Aldehydes	456.08 ± 77.6 ^a^	147.14 ± 51 ^b^	159.53 ± 5.90 ^b^	456.77 ± 2 ^a^	182.59 ± 17.7 ^b^
Esters	75.04 ± 20.3 ^b^	70.06 ± 7.72 ^b^	35.97 ± 14.06.71 ^c^	117.68 ± 9.7 ^a^	44.6 ± 1.3 ^bc^
Alcohols	385.67 ± 259.1 ^a^	158.73 ± 50.17 ^a^	269.45 ± 111 ^a^	273.2 ± 10.6 ^a^	83.31 ± 1.2 ^a^
Ketones	44.84 ± 12.2 ^b^	51.97 ± 14.19 ^b^	125.18 ± 49.13 ^a^	86.36 ± 9 ^ab^	42.17 ± 20.2 ^b^
Acids	630.34 ± 149.2 ^ab^	476.18 ± 19.82 ^ab^	588.29 ± 120.52 ^ab^	742.04 ± 11 ^a^	401.23 ± 117.2 ^b^
Pyrazines	16.23 ± 6.61 ^bc^	9.53 ± 7.24 ^c^	70.61 ± 28.51 ^a^	56.92 ± 20.51 ^ab^	20.57 ± 12.6 ^bc^
Terpenes	10.79 ± 2.20 ^a^	8.48 ± 3.32 ^a^	4.28 ± 3.34 ^ab^	7.59 ± 2.4 ^ab^	1.73 ± 0.9 ^b^
Others	13.14 ± 6.63 ^b^	4.83 ± 2.79 ^b^	5.02 ± 0.42 ^b^	46.08 ± 9.1 ^a^	2.13 ± 0.1 ^b^

The superscript letters indicate significant differences between the volatile compound concentrations of cocoa bean samples from AF systems in relation to cocoa production areas according to the Fischer test at *p* < 0.05.

**Table 3:** Changes in total concentration of desirable sensory compounds of dry fermented cocoa beans as a function of cropping system in different cocoa-producing regions of Côte d’Ivoire.

Useful Sensory Compounds	Kovats Index (NIST)	Calculated Kovats Index	Odor Description	Cropping System	Concentration of Sensory Compounds of Cocoa Beans from PRODUCING regions (µg.g^−1^)				
					Adz	Agni	Divo	Guib	Méa
3-Methylbutyl acetate	1123	1123	Fruity, banana	AF	47.7 ± 17 ^b^	2.2 ± 0.1 ^c^	2.8 ± 1.2 ^c^	84.2 ± 3.7 ^a^	15.8 ± 2.9 ^c^
				FS	75.2 ± 2.9 ^a^	37.2 ± 9.1 ^ab^	33.2 ± 36.3 ^ab^	41.9 ± 17 ^ab^	18.6 ± 9.4 ^b^
2-Methylbutyl acetate	1125	1120	Fruity, banana	AF	72.6 ± 1.8 ^a^	24.4 ± 5.6 ^b^	9.5 ± 1.3 ^b^	76.3 ± 11.8 ^a^	16.0 ± 3.6 ^b^
				FS	75.2 ± 2.9 ^a^	37.2 ± 9.1 ^ab^	33.2 ± 36.3 ^ab^	39.9 ± 13 ^ab^	18.6 ± 9.4 ^b^
Benzyl acetate	1720	1714	Sweet, floral, fruity	AF	1.4 ± 0.2 ^a^	0.8 ± 0.2 ^a^	1.7 ± 1.2 ^a^	3.4 ± 2.3 ^a^	0.5 ± 0.1 ^a^
				FS	2.8 ± 1.1 ^a^	1.8 ± 1.5 ^a^	2.5 ± 0.4 ^a^	1.5 ± 0.8 ^a^	1.8 ± 1.5 ^a^
2-Methylpropanal	819	823	Chocolate	AF	0.5 ± 0.1 ^c^	2.1 ± 0.2 ^a^	1.5 ± 0.1 ^ab^	1.3 ± 0.3 ^abc^	0.9 ± 0.7 ^bc^
				FS	2.5 ± 0.3 ^ab^	3.1 ± 1.3 ^a^	0.9 ± 0.5 ^b^	1.5 ± 0.6 ^ab^	1.1 ± 0 ^b^
2-Methylbutanal	914	872	Cocoa, chocolate, almond	AF	8.2 ± 9.9 ^b^	1.5 ± 1.1 ^b^	3.7 ± 3.3 ^b^	65.3 ± 3.3 ^a^	2.4 ± 0.1 ^b^
				FS	3.2 ± 1.3 ^a^	1.8 ± 1 ^a^	1.7 ± 0.6 ^a^	26.3 ± 9.7 ^a^	1.7 ± 0.2 ^a^
3-Methylbutanal	918	876	Cocoa, chocolate	AF	1.4 ± 0.2 ^b^	1.1 ± 0.3 ^bc^	1.1 ± 0.2 ^bc^	2.0 ± 0.1 ^a^	0.8 ± 0.1 ^c^
				FS	1.6 ± 0.8 ^a^	0.94 ± 0.1 ^a^	1.4 ± 0.3 ^a^	0.7 ± 0.3 ^a^	1.3 ± 0.1 ^a^
Benzyl alcohol	1870	1878	Floral, pink, phenolic	AF	3.9 ± 1.6 ^a^	2.7 ± 0.2 ^a^	4.6 ± 2.7 ^a^	5.7 ± 2.5 ^a^	3.2 ± 0.1 ^a^
				FS	5.6 ± 1.1 ^a^	5.1 ± 0.9 ^a^	5.1 ± 0.6 ^a^	3.2 ± 1.2 ^a^	6.4 ± 3.1 ^a^
2-Phenylethanol	1907	1907.8	Floral, Flowery	AF	45.0 ± 21. 2^a^	65 ± 2.1 ^a^	23.7 ± 12.6 ^a^	75.4 ± 54.3 ^a^	40.6 ± 6.8 ^a^
				FS	28.8 ± 13.8 ^b^	102.9 ± 2.1 ^a^	19.0 ± 2.7 ^b^	22.7 ± 8.2 ^b^	29.0 ± 6.9 ^b^
Acetoin	1285	1305	Creamy, buttery	AF	30.1 ± 9.6 ^b^	34.4 ± 11.2 ^b^	93.1 ± 36.4 ^a^	57.3 ± 4.9 ^ab^	33.3 ± 19.2 ^b^
				FS	57.4 ± 27.2 ^bc^	25.6 ± 6.9 ^c^	99.3 ± 4.3 ^ab^	116.5 ± 7.6 ^a^	71.5 ± 22.4 ^b^
2-Acetoxybutan-3-one	1378	1405	Sweet, creamy, buttery	AF	1.9 ± 1.3 ^b^	2.5 ± 1.1 ^b^	14.4 ± 9.1 ^a^	6.1 ± 2.1 ^ab^	1.1 ± 0.7 ^b^
				FS	2.7 ± 1.4 ^c^	1.2 ± 0.9 ^c^	19.3 ± 0.2 ^a^	10.4 ± 4.2 ^b^	3.7 ± 3.1 ^c^
Acetophenone	1647	1624	Flowery, sweet	AF	5.2 ± 0.4 ^a^	6.7 ± 1.9 ^a^	4.8 ± 0.9 ^a^	4.4 ± 1.3 ^a^	3.7 ± 1.3 ^a^
				FS	4.8 ± 0.1 ^b^	6.7 ± 1 ^a^	4.4 ± 0.5 ^b^	3.4 ± 0.5 ^b^	3.53 ± 0.5 ^b^
2.3.5-Trimethylpyrazine	1402	1407	Cocoa, roasted, baked, Peanut, roasted	AF	2.06 ± 0.7 ^bc^	1.1 ± 0.5 ^c^	4.3 ± 1.1 ^a^	3.5 ± 1.1 ^ab^	2.2 ± 0.4 ^bc^
				FS	2.3 ± 1.1 ^bc^	1.1 ± 0.6 ^c^	4.1 ± 0.2 ^a^	2.7 ± 0.7 ^ab^	4 ± 0.3 ^a^
Tetramethylpyrazine	1469	1468	Milk coffee, roasted, chocolate	AF	14.1 ± 5.9 ^bc^	8.4 ± 6.7 ^c^	66.2 ± 27.4 ^a^	53.4 ± 19. 5^ab^	18.5 ± 12.2 ^bc^
				FS	14.68 ± 7.8 ^bc^	3.61 ± 2.3 ^c^	64.8 ± 15.9 ^ab^	78.47 ± 40.1 ^a^	60.2 ± 18 ^ab^
Cis-linalool oxide	1444	1473.3	Sweet, floral, earthy, woody	AF	1.4 ± 0.2 ^a^	1.05 ± 0 ^ab^	0.7 ± 0.2 ^b^	1.3 ± 0.5 ^ab^	0.6 ± 0.1 ^b^
				FS	1.45 ± 0.2 ^a^	1.2 ± 0.2 ^ab^	0.63 ± 0 ^c^	1.2 ± 0.3 ^ab^	0.8 ± 0.1 ^bc^
Linalool	1547	1548.4	Floral, rose, sweet, green, citrus	AF	2.4 ± 0.4 ^a^	2.4 ± 1 ^a^	0.8 ± 0.5 ^b^	1.1 ± 0.5 ^ab^	0.3 ± 0.1 ^b^
				FS	2.2 ± 0.2 ^ab^	3.4 ± 1 ^a^	0.5 ± 0.1 ^c^	1.00 ± 0.2 ^bc^	0.5 ± 0.2 ^c^

AF = agroforestry, FS = full sun. The superscript letters indicate significant differences between some desirable volatile compound concentrations of cocoa bean samples from AF systems and cocoa bean samples based on cocoa production areas according to the Fischer test at *p* < 0.05.

## Data Availability

The original contributions presented in the study are included in the article, further inquiries can be directed to the corresponding author.
